# Recurrent and Non-Recurrent Mutations of *SCN8A* in Epileptic Encephalopathy

**DOI:** 10.3389/fneur.2015.00104

**Published:** 2015-05-15

**Authors:** Jacy L. Wagnon, Miriam H. Meisler

**Affiliations:** ^1^Department of Human Genetics, University of Michigan, Ann Arbor, MI, USA

**Keywords:** sodium channel, epilepsy, seizures, SUDEP, CpG, Dravet syndrome, mutation

## Abstract

Mutations of the voltage-gated sodium channel *SCN8A* have been identified in approximately 1% of nearly 1,500 children with early-infantile epileptic encephalopathies (EIEE) who have been tested by DNA sequencing. EIEE caused by mutation of *SCN8A* is designated EIEE13 (OMIM #614558). Affected children have seizure onset before 18 months of age as well as developmental and cognitive disabilities, movement disorders, and a high incidence of sudden death (SUDEP). EIEE13 is caused by *de novo* missense mutations of evolutionarily conserved residues in the Na_v_1.6 channel protein. One-third of the mutations are recurrent, and many occur at CpG dinucleotides. In this review, we discuss the effect of pathogenic mutations on the structure of the channel protein, the rate of recurrent mutation, and changes in channel function underlying this devastating disorder.

## Introduction

Neuronal voltage-gated sodium channels contain one large, pore-forming α subunit and two smaller β subunits (1). They regulate cellular excitability by controlling the flow of sodium ions across the cell membrane. The sodium channel α subunit genes *SCN1A*, *SCN2A*, and *SCN8A* are broadly expressed in brain neurons, where they play a critical role in the regulation of neuronal excitability. Mutations in each of these closely related genes can result in early-infantile epileptic encephalopathy (EIEE), characterized by early-onset seizures that are refractory to treatment and accompanied by cognitive and behavioral disabilities (2, 3). Mutations of *SCN1A* are responsible for EIEE6 (Dravet syndrome). *SCN2A* mutations have been identified in EIEE11 (Ohtahara syndrome). Since 2012, more than 60 mutations of *SCN8A* have been identified in EIEE13 (OMIM #614558). In this article, we review the emergence of *SCN8A* as an important cause of EIEE.

*SCN8A* encodes the voltage-gated sodium channel Na_v_1.6, the major sodium channel at nodes of Ranvier (4) and at the axon initial segment (AIS) of excitatory and inhibitory neurons (5–7). At the AIS, Na_v_1.6 regulates the initiation of action potentials (8), and at nodes of Ranvier it contributes to nerve conduction velocity (9). The effects of loss of Na_v_1.6 have been studied in spontaneous mouse mutants. Partial or complete loss of Na_v_1.6 in mice causes reduced neuronal excitability as well as tremor, ataxia, and dystonia. Null mice develop hindlimb paralysis and death by 3 weeks of age (3). Heterozygous carriers of loss-of-function mutations in the mouse exhibit only mild abnormalities including disrupted sleep architecture and elevated anxiety (10, 11). On some strain backgrounds, heterozygous null mice exhibit spike–wave discharges, the hallmark of absence (non-convulsive) epilepsy, without spontaneous convulsive seizures (12).

## Discovery of Patient Mutations in *SCN8A*

The role of *SCN8A* in human disease was first examined by a candidate gene approach in families segregating inherited movement disorders that resembled the defects in mutant mice. One family with a mutation of *SCN8A* was identified by this approach. The proband had ataxia and intellectual disability and was found to be heterozygous for a protein truncation mutation of *SCN8A* (13). Three additional heterozygous family members had intellectual disabilities without ataxia. It is important to note that none of the heterozygous null individuals in this family had a history of seizures, indicating that simple loss of *SCN8A* function is not epileptogenic. A screen of 80 patients with familial essential tremor failed to detect *SCN8A* variants (14). Analysis of families with inherited ataxia was also negative (Meisler, unpublished observations).

The recent introduction of exome sequencing in patients with early-onset epileptic encephalopathy resulted in identification of *de novo* mutation of *SCN8A* as an important cause of this non-familial disorder. The first *de novo* mutation was found in a patient with seizure onset at 6 months of age (15). More than 60 *de novo* mutations of *SCN8A* have since been identified by exome and genome sequencing and, more recently, by inclusion of *SCN8A* in commercial epilepsy gene sequencing panels (16–28).

Four studies utilized targeted sequencing strategies to identify pathogenic variants in large cohorts of epileptic encephalopathy patients. The proportion of EIEE cases with mutations in *SCN8A* was 3/500 (17), 2/264 (18), 7/683 (25), and 1/110 (26). Thus, mutations in *SCN8A* account for close to 1% of epileptic encephalopathy.

## Shared Clinical Features of EIEE13 Patients

Most patients with EIEE13 have seizure onset between birth and 12 months of age, with a median age of 4 months. Several patients exhibited seizures within a week or two of birth. Generalized tonic and tonic–clonic seizures are present in most patients. Atonic, myoclonic, and focal and absence seizures are not uncommon. Febrile seizures are rare in EIEE13, providing a useful distinction between EIEE13 and Dravet syndrome. Prior to seizure onset, development is normal in approximately half of patients. After seizure onset, developmental regression often results in mild to severe intellectual disability. A distinguishing feature of EIEE13 is the movement disorders, which range from mild ataxia to choreoathetosis and, in several patients, quadriplegia. Hypotonia and hypertonia are frequent, sometimes in the same patient. SUDEP has been reported in 5/40 patients.

EEG at or near the time of seizure onset is normal in approximately 50% of cases. In the following months, most individuals develop EEG abnormalities including focal or multi-focal sharp waves or spikes, moderate to severe background slowing, and spike–wave complexes. Hypsarrhythmia is rare.

MRI scans usually appear normal at onset of EIEE13. After seizure onset, the MRI remains normal in approximately 50% of patients. The most common MRI abnormality is mild diffuse atrophy or mild to moderate cerebral atrophy. Cerebellar atrophy and thinning of the corpus callosum has also been reported. The extent of cerebral and cerebellar atrophy appears to be correlated with severity of the movement disorder. Under-developed cortex and defective myelination has been seen in a few cases. Microcephaly is rare.

## Drug Responses in Patients with EIEE13

Seizures in EIEE13 patients are typically refractory to treatment, but up to 1/3 of patients have enjoyed seizure-free periods. Approximately, 40% of patients for whom we have detailed clinical histories have responded favorably to anticonvulsants that directly modulate sodium channel activity (“sodium channel blockers”) including valproic acid, phenytoin, carbamazepine, oxcarbazepine, lamotrigine, and topiramate (Table 1). Monotherapy with sodium channel blockers has been effective for some patients. Sodium channel blockers have been effective in patients with either gain-of-function or apparent loss-of-function mutations. Carbamazepine and its derivative oxcarbazepine have been useful in the largest number of patients. Patients carrying the identical *SCN8A* mutation can differ in their drug responses, indicating a significant role for modifier genes and/or stochastic developmental processes (Tables 1 and 2).

**Table 1:** **Reported drug response in epileptic encephalopathy due to *SCN8A* mutations**.

Amino acid substitution	Channel domain	Effect on function	Effective treatment	Seizure response	Reference
p.Val216Asp	DIS3-4		**VPA**	Seizure control	(23)
p.Leu407Phe	DIS6		**CBZ**	75% reduction	(53)
p.Phe846Ser	DIIS4		**PHT**, **LTG**, PB KD, VNS	Temporarily effective	(53)
p.Ala890Thr	DIIS5		**VPA**	Seizure control	(25, 53)
			**VPA, OXC**	
p.Asn984Lys	Near DIIS6	GOF	**PHT**, **ZNS**, PB LEV, CLB	Seizure control	(24)
p.Ile1327Val	DIIIS4-5		High **PHT**	Temporarily effective	(27)
p.Gly1451Ser	DIIIS6	LOF	**CBZ**	Seizure control	(24)
p.Asn1466Lys	Inactivation gate		**PHT**, **TPM**, GBP, ACTH, MDL, LD	Temporarily effective	(23)
p.Asn1466Thr	Inactivation gate		**TPM**, LEV	Seizure control	(23)
p.Val1592Leu	DIVS3		**OXC**	Seizure control	(25)
p.Ser1596Cys	DIVS3		**OXC**, **LTG**, PB, LEV	75% reduction	(53)
p.Ile1605Arg	DIVS3		**CBZ**	Seizure control	(25)
p.Arg1617Gln	DIVS4		**CBZ**	Temp. effective	(23, 53)
			**OXC**	Seizure control	
p.Ala1650Thr	DIVS4-5		**CBZ**, **TPM**	Seizure control	(23)
p.Asn1768Asp	DIVS6	GOF	**VPA**, **LTG**, CLB	Temporarily effective	(15)
p.Arg1872Trp	C-term		**TPM**, **LCM**, LEV, VGB, KD	Febrile breakthrough	(23)

*ACTH, adrenocorticotropic hormone; CBZ, carbamazepine; CLB, clobazam; GBP, gabapentin; KD, ketogenic diet; LCM, lacosamide; LD, lidocaine; LEV, levetiracetam; LTG, lamotrigine; MDL, midazolam; OXC, oxcarbazepine; PB, phenobarbital; PHT, phenytoin; TPM, topiramate; VGB, vigabatrin; VNS, vagal nerve stimulator; VPA, valproic acid; ZNS, zonisamide. Drugs classified as sodium channel blockers are indicated in bold*.

**Table 2:** **Phenotypic variability in patients with the p.Arg1617Gln mutation in *SCN8A***.

Patient	Age of onset	Seizure type	Developmental progression	EEG	Drug response	Reference
1	3 months	Febrile seizures, tonic–clonic	Sat, crawled 24 months	Normal at 3 months	CBZ temp. effective	(23)
2	5.5 months	Generalized tonic–clonic, tonic, clonic, myoclonic, atypical absence	Hypotonia, wheelchair-bound, no speech, SUDEP at 3 years	Bilateral delta slowing and left temporal spikes and/or slow waves	Refractory	(25)
3	6 months	Generalized tonic	Head control 7 months., sat 12 months	Normal at 11 months, spike and spike–wave complexes in left rolandic at 3 years	OXC effective	(53)
4	7 months	Generalized tonic–clonic	Spasticity, sat 8 months, walked 17 months, speech 24 months	Not described	No details	(16)
5	12 months	Generalized tonic–clonic	Not described	Suggested anterior midline-frontal onset	Refractory	(21)

The limited clinical evidence suggests that sodium channel blockers may be the best first line treatments for patients with *SCN8A* mutations, rather than modulators of GABA signaling or other synaptic function. By contrast, sodium channel blockers usually exacerbate seizures in patients with Dravet syndrome, which is caused by a deficiency of *SCN1A* activity. EIEE13, which is due to overactivity of *SCN8A*, has not been reported to be exacerbated by sodium channel blockers. Seizure exacerbation in EIEE13 patients has been observed with administration of levetiracetam. The ketogenic diet is reported to be effective for as many as 1/3 of patients with Dravet Syndrome. The response of patients with EIEE13 is not yet known. Early diagnostic DNA sequencing to distinguish between EIEE13 and Dravet Syndrome is thus very important for the selection of treatment.

## Recurrent Mutations of *SCN8A*

**Figure 1:**
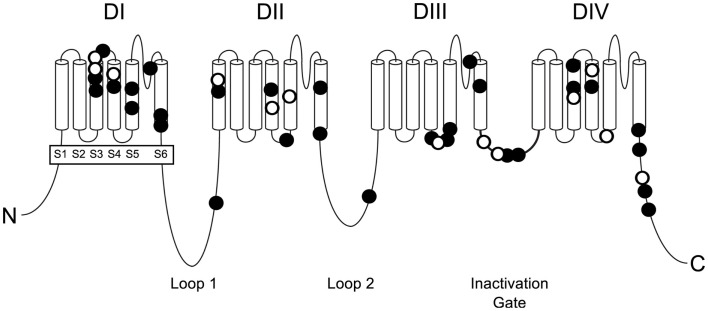
**Positions of missense mutations of *SCN8A* in epileptic encephalopathy**. The four homologous domains of Na_v_1.6 (DI to DIV) each contain six transmembrane segments (S1 to S6). The large inter-domain cytoplasmic loops 1 and 2 are evolutionarily less well-conserved than the transmembrane and linker domains. Loop 3 functions as the inactivation gate and is very highly conserved. Closed symbols, EIEE13 mutations in a single patient; open symbols, recurrent mutations.

The known mutations of *SCN8A* in EIEE13 are missense mutations that arose *de novo* in the patient or, in two cases, were inherited from a mosaic parent. All of the missense mutations result in substitution of an evolutionarily conserved amino acid residue. The locations of these mutations are shown in Figure 1. One-third of the mutations have reoccurred in two or more unrelated individuals (Figure 1, open circles). The molecular alterations in the recurrent mutations are indicated in Table 3. There have been five occurrences of the missense mutation p.Arg1617Gln and nine mutations of arginine residue 1872, four with substitution of glutamine, three with substitution of tryptophan, and two with substitution of leucine.

**Table 3:** **Recurrent *de novo* mutations of *SCN8A* in epileptic encephalopathy**.

Amino acid substitution	Nucleotide substitution	Exon	Channel domain	CpG	#	Reference
p.Asn215Asp	c.643 A > G	6A	DIS3	No	2	DECIPHER 2015 (25)
p.Ala890Thr	c.2668 G > A	16	DIIS5	No	2	(25, 53)
p.Ile1327Val	c.3979 A > G	22	DIIIS4-5	No	2	(19, 27)
p.Arg1617Gln	c.4850 G > A	27	DIVS4	Yes	5	(16, 21, 23, 25, 53)
p.Ala1650Thr	c.4948 G > A	27	DIVS4-5	No	2	(23, 25)
p.Arg1872Trp	c.5614 C > T	27	C-term	Yes	9	(17, 23, 25), unpublished obs.
p.Arg1872Gln	c.5615G > A	
p.Arg1872Leu	c.5615 G > T					

**Figure 2:**
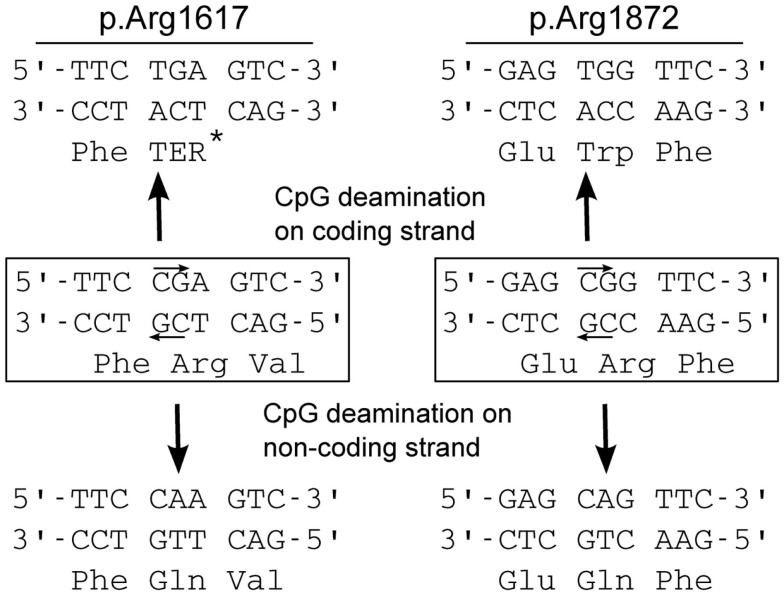
**Mechanism for recurrent mutation of *SCN8A* at CpG dinucleotides**. Arginine 1617 is encoded by a CGA codon, and arginine 1872 is encoded by a CGG codon. Both contain CpG dinucleotides on coding and non-coding strands (small arrows). Cytosine methylation followed by deamination on either strand leads to mutation: at arginine 1617, replacement by the premature termination codon (TGA, Ter) or glutamine (Gln); at arginine 1872, replacement by tryptophan (Trp) or glutamine (Gln). The premature termination mutation p.Arg1617Ter has not been seen in EIEE13 patients, and would probably not cause seizures (see text).

One-third of the recurrent mutations are located at CpG dinucleotide residues, e.g., in arginine codons 1872 (CGG) and 1617 (CGA). CpG dinucleotides are mutation hotspots that can undergo enzymatic methylation of cytosine followed by spontaneous deamination of the methylated C to form thymine (Figure 2). Both of these arginine codons contain CpG sequences on coding and non-coding strands, and CpG deamination at these sites can account for most of the observed amino acid substitutions (Figure 2).

Clinical information is available for five patients with the p.Arg1617Gln mutation. Remarkably, these patients differ in many respects, including age of seizure onset, seizure type, severity of co-morbidities, and drug response (Table 2). These observations emphasize the important role of genetic background, including variation in other ion channel genes, on the clinical outcome for any individual patient.

Recurrent mutations of *SCN1A* have also been observed in Dravet syndrome. Recurrent mutations account for 25% of pathogenic mutations, and 1/3 of recurrent mutations are located at CpG dinucleotides (29). Recurrent deletions of short direct repeats that are susceptible to slipped-strand mispairing account for generation of some null alleles of *SCN1A*.

Sodium channels are among the most highly conserved proteins in the mammalian genome. *SCN8A* ranks among the 2% of human proteins that are least tolerant of variation [see dataset S2 in Ref. (30)]. The pathogenic missense mutations of *SCN8A* identify a subset of residues whose substitution results in elevated channel activity.

## Location and Functional Consequences of *SCN8A* Mutations

**Figure 3:**
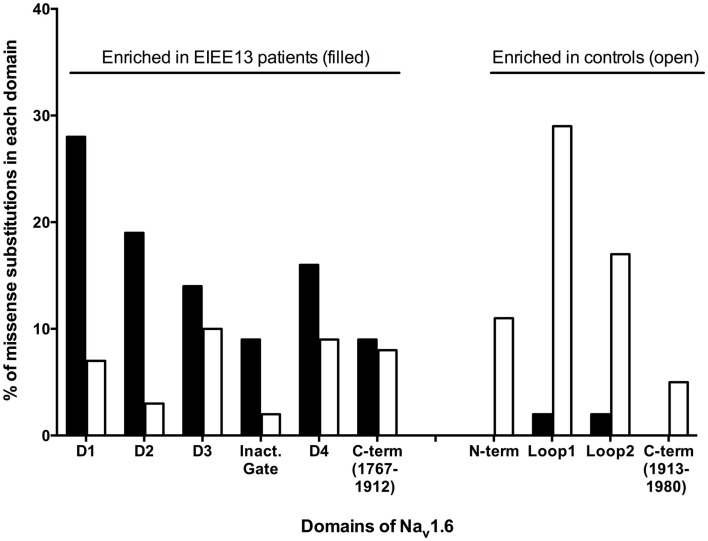
**Differential distribution of pathogenic and non-pathogenic variants in the protein domains of *SCN8A***. Mutations of patients with EIEE13 are compared with variation in the ExAC database (exac.broadinstitute.org, accessed 04/2015), which excludes severe pediatric disease. Each amino acid substitution was counted once. Data were available for 43 mutations in EIEE13 patients and 228 variants from the ExAC database. Filled bars, EIEE13 patients; open bars, ExAC controls; Inact. gate, inactivation gate.

Sodium channel α subunits contain four homologous domains (DI-DIV), each with six highly conserved transmembrane segments designated S1–S6 (Figure 1). Less-conserved cytoplasmic loops separate domains DI and DII, and domains DII and DIII. Domains III and IV are separated by a short, highly conserved inactivation gate (Figure 1). The proximal 2/3 of the C-terminus (residues 1767–1912) harbors several binding sites for interacting proteins, including sodium channel beta subunit β1 (31), intracellular FGFs (32–34), and calmodulin (35). The final 1/3 of the C-terminus, downstream of the calmodulin-binding motif, is disordered and not well-conserved. Most of the mutated residues in EIEE13 are located in the highly conserved portions of the protein: the domains containing transmembrane segments, inactivation gate, and proximal 2/3 of the C-terminus of Na_v_1.6 (Figure 3). In contrast, the non-pathogenic variants reported in the ExAC database of more than 60,000 exomes [Exome Aggregation Consortium, Cambridge, MA, USA (URL: http//exac.broadinstitute.org), accessed 04/2015] are concentrated in the non-conserved cytoplasmic loops of the channel (Figure 3).

Five mutations of *SCN8A* have been tested in functional assays in transfected cells (Table 4). Three of these have dramatic gain-of-function effects. p.Asn1768Asp causes impaired channel inactivation and elevated persistent sodium current (15). As predicted by these experiments, transfection of primary hippocampal neurons with the mutant Na_v_1.6 cDNA increases neuronal excitability and promotes spontaneous firing (15). Similar missense mutations causing increased persistent current have also been identified in three other neuronal sodium channels. *SCN1A* mutations with increased persistent current were observed in patients with generalized epilepsy with febrile seizures plus (GEFS+) and Dravet Syndrome (36–38). *SCN2A* mutations with delayed inactivation and increased persistent current were described in both human and mouse (39–43). An *SCN3A* mutation with increased persistent current was identified in a child with pediatric partial epilepsy (44, 45). Thus, increased persistent current is a common pathological mechanism in epilepsy due to sodium channel mutations.

**Table 4:** **Functional effects of five *SCN8A* mutations in patients with epileptic encephalopathy**.

Amino acid substitution	Channel domain	Cell assay	Effect on function	Reference
p.Arg223Gly	DIS4	HEK	Partial LOF, protein unstable at 37°C	(20)
p.Thr767Ile	DIIS1	ND7/23	GOF, hyperpolarizing shift in activation voltage	(22)
p.Asn984Lys	Near DIIS6	HEK	GOF, hyperpolarizing shift in activation voltage	(24)
p.Gly1451Ser	DIIIS6	HEK	LOF, no activity at 37°C	(24)
p.Asn1768Asp	DIVS6	ND7/23	GOF, increased persistent current	(15)

Two additional *SCN8A* mutations, p.Thr767Ile and p.Asn 984Lys, cause a hyperpolarized shift in the voltage-dependence of activation, which is predicted to result in premature neuronal firing (22, 24). Hyperpolarized shifts in activation have also been observed in missense mutations of *SCN1A* and *SCN2A* that cause early-onset epilepsy (46–48). Elevated activity of Na_v_1.6 thus appears to be the cause of seizures in these two cases.

The two mutations of *SCN8A* with apparent loss-of-function in transfected cell assays are puzzling. Simple loss-of-function mutations caused by protein truncation do not cause seizures in human or mouse (3, 13). It seems most likely that these two patient mutations actually exhibit gain-of-function *in vivo*. p.Gly1451Ser encodes a stable protein that lacks channel activity in transfected cells (24). p.Arg223Gly, results in protein instability at 37°C, greatly reducing current density but at 30°C, partial function is restored (20). At 30°C, activation and inactivation kinetics do not differ from wildtype but the mutant channel generates elevated ramp current in response to slow ramp depolarizations (20). Increased ramp current is a gain-of-function property that has been observed in a mutation of Na_v_1.3 in pediatric partial epilepsy (44, 45). Because the p.Arg223Gly mutation affects the second arginine residue in the DIS4 voltage sensor, it may also cause increased gating pore current, as observed for pathogenic mutations of Na_v_1.4 in periodic paralysis and Na_v_1.5 in dilated cardiomyopathy (49, 50). Thus, these two apparently inactive channel proteins may exhibit gain-of-function features *in vivo*. Thorough analysis of additional patient mutations will be required for definition of the relationship between genotype and phenotype of missense substitutions in *SCN8A*.

## Modeling *SCN8A* Epileptic Encephalopathy in the Mouse

The earliest models of *Scn8a* dysfunction in the mouse had partial or complete loss of channel activity, and did not exhibit seizures (3). To generate a mouse model of EIEE13, we introduced the patient mutation p.Asn1768Asp into the mouse genome (51). Heterozygous *Scn8a^N1768D/^*^+^ mice recapitulate several features of EIEE13, including spontaneous convulsive seizures, ataxia, and sudden death (52). In heterozygous mutant mice, seizure onset occurs at 2–4 months of age. The mice may have several generalized tonic–clonic seizures per day, and progress to sudden death within 1 month of seizure onset.

Like many patients, the heterozygous *Scn8a^N1768D/^*^+^ mice have normal EEG prior to seizure onset (52). After seizure onset, they exhibit sporadic, semiperiodic biphasic slow wave or sharp wave–slow wave complexes. In addition, there are multiple daily interictal epileptiform discharges with diffuse polyspikes or single spike–wave discharges, similar to those in EIEE13. These discharges are frequently accompanied by myoclonic jerks or spasms. Unlike the patients, the mice do not demonstrate impaired learning or behavior, brain atrophy, or structural abnormalities of the brain. This novel *Scn8a* mouse model will be useful for investigation of pathogenesis and mechanisms of SUDEP. It will also be useful for evaluation of new therapies.

## Conclusion

Large-scale sequencing of patient exomes has revealed the role of *SCN8A* in human epilepsy. Mutation of *SCN8A* accounts for approximately 1% of EIEE. The predominance of *de novo* mutations in children with EIEE13 reflects the severity of the disorder, which prevents transmission to the next generation. One-third of the mutations in EIEE13 are recurrent, and most occur in highly conserved regions of the channel. Further analysis of the functional effects of additional *SCN8A* mutations will be required to generate a comprehensive view of structure/function relationships. Functional differences among pathogenic mutations of *SCN8A* will have implications for selection of treatment for individual patients and for development of new therapies.

## Conflict of Interest Statement

The authors declare that the research was conducted in the absence of any commercial or financial relationships that could be construed as a potential conflict of interest.
